# Gene therapy for spinal muscular atrophy: the benefit–cost profile

**DOI:** 10.1016/j.eclinm.2021.101065

**Published:** 2021-07-27

With 1 in 10 000 babies affected by spinal muscular atrophy, approval of the first gene therapy by the UK National Institute of Health and Care Excellence (NICE) in March, 2021, brings hope for a cure. Spinal muscular atrophy causes progressive loss of muscle function, respiratory issues, and reduced mobility, with symptoms appearing in the first 6 months of life. Untreated type 1 disease, the most severe form, accounts for 60% of cases and is the leading genetic cause of mortality in babies, resulting in short life expectancy of 2 years for 90% of untreated patients. Until 2016, there was no treatment for spinal muscular atrophy, since then, three drugs became available. In May, 2021, an infant aged 5 months became the first patient in the UK to receive onasemnogene abeparvovec (branded as zolgensma), a one-off gene therapy that became available through the National Health Service. This drug, classed as the most expensive in the world, has sparked global controversy with an extortionate price of £1.79 million per dose.

Onasemnogene abeparvovec is a one-time intravenous injection introducing the *SMN1* transgene into motor neurons using an adeno-associated viral vector to promote SMN protein expression and replace the non-functional *SMN1* gene, with the hope to slow down disease progression. Motor neurons do not divide; therefore, one injection is expected to have long-term stability. However, the injected transgene might eventually be lost.

The highest incidence of spinal muscular atrophy is seen in Central and Eastern Europe. In the USA, where approximately 25 000 children live with the disorder, onasemnogene abeparvovec received fast-track approval by the US Food and Drug Administration for patients younger than 2 years in May, 2019. The European Medicines Agency then approved the drug in May, 2020, under a conditional marketing authorisation (along with NICE) to be reviewed regularly when long-term data become available. NICE guidelines were published on July 7, 2021, recommending the use of onasemnogene abeparvovec in the UK for infants aged 6 months or younger with spinal muscular atrophy type 1 requiring daily permanent ventilation and for pre-symptomatic patients with a biallelic mutation in *SMN1* and up to three copies of *SMN2*. Approximately 65–80 of 100 infants born in the UK with the disease each year are expected to benefit from this treatment.

The NICE guidelines are based on pooled analysis from two published studies with a follow-up of 18–24 months in 37 patients. The START and STR1VE-US studies showed that 94% of patients did not require permanent ventilation at study completion and 68% patients were able to sit independently for 30 seconds or more. 15 of 34 patients had treatment-related adverse events, which were resolved during the studies. The most common adverse events were vomiting and increased hepatic aminotransferase. Further studies support these results; in STR1VE-EU, 43% patients could sit independently for 30 seconds or longer and in SPR1NT, all 14 babies were alive at 14 months and did not require ventilatory support, compared with 26% in the untreated natural history cohort.

Interim data were extrapolated to estimate long-term 5-year efficacy from the LT‑001 trial (NCT03421977), a 15-year follow-up of START. Ten of 13 babies were alive at median time 5.2 years since treatment without needing permanent ventilation and all maintained or gained developmental milestones achieved at the end of START. However, serious adverse events such as acute respiratory failure and pneumonia occurred in eight patients. Data for type 2 and 3 disease, which occurs later in life with less severe symptoms, are not yet available.

In the USA, nusinersen, the first therapy for spinal muscular atrophy approved in 2016, costs $750 000 in the first year and $375 000 every following year for a patient's lifetime compared with onasemnogene abeparvovec, which costs $2.1 million for a one-off injection in five $425 000 instalments. How is this high cost justified? Novartis used a standard value-based pricing model for onasemnogene abeparvovec, which sets the price based on how much the customer believes a drug is worth. The actual costs are unknown; however, on average, drug development costs $2 billion. Early development of this orphan drug was funded by the National Institutes of Health (NIH) and various charities, thus, much of this cost was subsidised. Large manufacturing costs for this highly specialised drug combined with a small number of sales and a one-time dose resulted in the high price. Additionally, the market size is small and because this disorder is rare, fewer infants can benefit, thus the price is increased to reach a return on investment.

Gene therapy is becoming increasingly integrated into medicine. Despite more than 1000 babies receiving onasemnogene abeparvovec and approval in 38 countries, the enormous price will exacerbate inequalities in access to medication between countries with publicly and privately funded health care but also for patients without any health care coverage. The justification of the high costs of onasemnogene abeparvovec is debateable, especially considering that the research was funded by taxpayer's money through NIH and other charities, and equal access must be ensured. Further questions around long-term effectiveness, maintenance of achieved motor milestones, and persistence of the transgene in motor neurons will be important to address in future studies.

*EClinicalMedicine*

